# Round the Hospitals

**Published:** 1920-12-25

**Authors:** 


### ROUND THE HOSPITALS.

The death of the Dowager Countess Roberts, widow of the great Field-Marshal, at the age of eighty-two, recalls to memory the splendid work she did in India in connection with the organisation of the nursing services for military hospitals. Her proposals to supplement the previously existing system of solely male nursing (orderlies) by trained women nurses were drawn up in 1886, and were accepted as an obligation of the State to be provided out of public funds. But she was not content with this important reform, and desired to provide for her nurses a means by which they could recruit their energies by visits to "the hills," to regain the strength and health so likely to be diminished on the plains. This obligation the State did not undertake, so Lady Roberts raised the necessary funds by a personal appeal. Later on she equipped also two wards for officers, staffed by nurses also paid for out of her fund. Thus the Commander-in-Chief's wife, as she then was, earned the gratitude of thousands of her countrymen and countrywomen in India, and inaugurated nursing reforms which the present generation should not allow to be forgotten; many women in her position might have been content to rely for their prestige on the brilliance of their husbands, but not so the late Countess.

In our issue of December 11 we announced the intention of the Minister of Labour to give a decision on the question whether the employment by a hospital supported out of voluntary contributions of trained sisters and nurses and probationer nurses is such employment as to bring them under the provisions of the Unemployment Insurance Act, and especially whether such employment is employment in domestic service. We are glad to find that the nursing profession and those interested in it are apparently bestirring themselves in this matter, for in the *London Gazette* of December 21 the announcement is made that an inquiry will be held at 8 Richmond Terrace, Whitehall, S.W., on Wednesday, January 5, 1921, at 11 A.M. The names and addresses of all persons wishing to attend the hearing must be received by the Principal Assistant Secretary, Ministry of Labour, Employment Department, Queen Anne's Chambers, Westminster, S.W. 1, not later than January 2, 1921. Failure to comply with this may entail refusal of a hearing. We hope that full advantage may be taken of this opportunity to put before the Minister the views of hospital committees, nurses, and nurses' associations.

The position of private and visiting nurses under the Unemployment Insurance Act seems to be governed by Clause (*h*) Part II. of the First Schedule, which excepts from the provisions of the Act persons engaged in employment otherwise than by way of manual labour and at a rate of remuneration exceeding in value two hundred and fifty pounds a year. The present rate of remuneration of private nurses working for a co-operation or on their own account, together with the value of emoluments (board, residence and washing), exceeds £250, and therefore such nurses are not insurable for unemployment any more than they are under the National Health Insurance Act.

The question as to what may be considered a fully-equipped staff for dealing with maternity and child-welfare work has been decided by the Southwark Borough Council. The borough has a population of about 210,000. At present the council have seven municipal centres for maternity and child-welfare work, three "wholly voluntary" centres, and four "partly municipal and partly voluntary" centres. In addition to attending at the municipal centres, the staff of health visitors and nurses have also to supervise the work of the other centres. To cope with this extensive work the council has decided that for the present the staff shall be increased to a lady superintendent health visitor, from six to nine health visitors, from four to five nurses, and a mothers' help. Two of the nurses would be specially set apart to deal with cases should there be epidemics of measles or influenza, but, except during such epidemics, they would be engaged on the general work. A weekly session is held at each centre, with a usual attendance of from seventy to one hundred mothers and children. At one centre alone at least 10 per cent. of the children are in the early stages of rickets or suffering from specific disease or some other complaint. All these suffering children require special treatment necessitating in most cases the daily visit from a nurse. When the maternity and child-welfare scheme has reached its full development Dr. Millsom, the medical officer of health, estimates it will cost the council £7,500 a year, half of which is recoverable from the Ministry of Health.

Camberwell Red Cross (King's College Hospital Division) has distinguished itself in a novel way. Mr. R. J. Coles, the hospital secretary, recently sent a message to headquarters inquiring whether any member of the detachment would submit to transfusion of blood to save the life of a patient. Three members instantly volunteered, and attended to have their blood graded, and the Commandant, Nurse Linstead, was selected, but before the operation could take place the patient died. Six members of the detachment have now given in their names to come forward for this purpose as and when they may be required. It is the first instance of the Red Cross organising such a service for the benefit of a large hospital. The loan depôt for nursing and surgical apparatus run by this division is proving a great boon to the neighbourhood, and we know of no better-organised Red Cross work in peace time than that which is being done in connection with King's College Hospital.

On Thursday, January 6, there is to be unveiled in the Church of St. Eizabeth a Memorial to nurses who died during the war. The service commences at 3.30 P.M., and the address will be given by the Right Rev. The Bishop of Croydon. After the service there will be a tea and reception. Invitations are not being sent out, but Miss Alsop and the Rev. A. Lombardini will welcome any who are desirous of being present at the unveiling ceremony at 3.30 P.M.

The father of a grateful patient lately had the happy thought to present to the Miller General Hospital the cup and saucer used by Florence Nightingale in the Crimea. The history of the cup and saucer is that they were brought from the Crimea by Corporal J. Nolan, Prince of Wales' Volunteers, which is now the South Lancashire Regiment. He gave them to his sister, who left them at her death to her son, the present donor. These relics, if the word is not too solemn, were among the objects displayed at the recent public inspection of the new out-patient department, which, till its conversion for immediate use, was the Greenwich Road Congregational Church and Schoolroom. The former church is now the waiting-hall. What was once the organ-loft is now a minor operating-room. The converted buildings will greatly add to the comfort of those attending for out-patient treatment.

The Trowbridge Hospital is entering on a new departure in taking over the work of the Nursing Association, which is now to be conducted from the hospital as headquarters. This is in some respects an excellent administrative change. It has been brought about by the resignation of Mrs. Fisher, the Secretary of the Nursing Association, and the impossibility of finding a successor. The doctors strongly approve of the new plan. The main difficulty to be apprehended is financial, but by keeping the accounts entirely distinct this should easily be overcome, and a more efficient nursing service provided. The arrangement is to be tried for twelve months as an experiment.

---

There was an interesting ceremony the other day at the Ministry of Pensions Hospital at Neath, when the regret of all at losing two of the nursing staff, owing to the expiry of their contracts, was warmly expressed. To Sister James the patients presented a silver travelling clock, books, and address. Dr. Vickery spoke in high praise of the Sister's work, and said that, though the address was signed only by the patients at present in the hospital, they were, he felt sure, representative of a very large number of South Wales heroes who had been nursed back to health and strength under her care. To Sister Southworth Major Alwyn Smith, D.S.O., F.R.C.S., the Cardiff orthopædic surgeon, presented a silver clock and address, in appreciation of her valued services in the operating theatre.

---

There is a very strong ambulance station at Plymouth, and steps are being taken to organise an efficient nursing auxiliary. The director has been fortunate enough to secure the services of Mrs. W. Pethybridge, a highly-qualified nursing sister, with, in addition, the C.M.B. certificate, to be matron-in-charge, and she is assisted by a small staff of efficient nurses who are ready to enlist volunteers. The need is for a body of nurses and aids sufficiently large to deal with the transport of invalids by ambulance, to attend at large public gatherings and render first-aid as required. It is hoped to form an auxiliary of ladies ready to lend their aid in institutions in times of emergency and to be at the service of the sick and infirm poor. The sisters and other officers have now been appointed, and classes for the instruction of the volunteers will shortly be started.

---

A curious debate took place at the meeting of the Chapel-en-le-Frith Guardians, when the charge-nurse sent in her resignation. Great efforts were made to induce the nurse to state her exact reasons for resigning, but, in view of the fact that the Press were present, we consider she acted wisely in refusing to do so. Cross-questioned persistently, she replied that it was not fair to ask her questions, as she "had her living to get." We can think of no more unsuitable way of dealing with resignations than this, and would point out that one of the main causes of unrest in Poor-Law institutions lies in the attempts so often made by Guardians to deal with the internal affairs of the institution at meetings held in public. One of the lady Guardians moved that a small committee be appointed to go into the matter, and this was agreed to.

---

Dr. Barnes presided at the enthusiastic annual meeting of the Carlisle Medical Auxiliary of the Church Missionary Society and gave some interesting particulars of the nursing needs of the Society. Carlisle has sent £100 a year to the cause for the past twenty-one years, and a fervent missionary spirit has been cultivated. The C.M.S. has increased the number of nurses in its employ from twenty-one in 1899 to seventy-one in 1919. Twenty-one doctors and sixteen nurses are at the present moment urgently required for work in Africa, Persia, India, and China.

---

The conversion of asylums into mental hospitals continues apace and means much more than a mere change of nomenclature. The report of Dr. Charles Planck, Medical Superintendent of the Brighton County Borough Mental Hospital, for the past year reveals that the nursing side in this class of hospitals has now assumed high importance. The percentage of recoveries amounted to 30.7 on the male and 30.0 on the female side. Taking both sexes together the death-rate was 19.0 per cent., largely owing to a severe outbreak of influenza, and the list of the other diseases which occurred among the patients is sufficient to demonstrate the need for general training in all who are called on to direct this branch of the work. We regret to learn that there is still much unrest among the younger members of the female nursing staff, with great difficulty in obtaining candidates. The working hours are sixty per week, including meal-times—far too long. By a curious misprint we learn that the nurses are allowed two and a-half days' holiday *a week*, instead of a month.

---

The Royal Southern Hospital, Liverpool, is among the winners in the race for a new Nurses' Home. We rejoice to hear that the extension for which appeal was made last March is now an accomplished fact, opened early in December, and so nearly paid for that the remainder must now be well on the way. Much more is at stake in this extension than better sleeping quarters. Its completion marks the beginning of many long-wished-for reforms which will put the Royal Southern definitely in the van of nursing progress.

---

At the final meeting of the Cavell Memorial Committee, held at the *Daily Telegraph* office, Viscount Burnham presided, and a financial statement was presented showing that the receipts amounted to £3,239 and the total cost to £3,627. The debit balance of £429 was contributed by the proprietors of the *Daily Telegraph*. Sir George Frampton said he had received large numbers of congratulatory letters on the Nurse Cavell Memorial from Italy, France, and Belgium. The President of the Milan Royal Academy had told him that in Italy it was considered one of the finest monuments in Europe.

